# Accuracy of Detecting Residual Disease After Cross Neoadjuvant Chemoradiotherapy for Esophageal Cancer (preSANO Trial): Rationale and Protocol

**DOI:** 10.2196/resprot.4320

**Published:** 2015-06-29

**Authors:** Bo Jan Noordman, Joel Shapiro, Manon CW Spaander, Kausilia K Krishnadath, Hanneke WM van Laarhoven, Mark I van Berge Henegouwen, Grard AP Nieuwenhuijzen, Richard van Hillegersberg, Meindert N Sosef, Ewout W Steyerberg, Bas PL Wijnhoven, J Jan B van Lanschot

**Affiliations:** ^1^Erasmus MC - University Medical Center RotterdamDepartment of SurgeryRotterdamNetherlands; ^2^Erasmus MC - University Medical Center RotterdamDepartment of GastroenterologyRotterdamNetherlands; ^3^Academic Medical CenterDepartment of GastroenterologyAmsterdamNetherlands; ^4^Academic Medical CenterDepartment of Medical OncologyAmsterdamNetherlands; ^5^Academic Medical CenterDepartment of SurgeryAmsterdamNetherlands; ^6^Catharina Cancer CenterDepartment of SurgeryEindhovenNetherlands; ^7^University Medical CenterDepartment of SurgeryUtrechtNetherlands; ^8^Atrium Medical CenterDepartment of SurgeryHeerlenNetherlands; ^9^Erasmus MC - University Medical Center RotterdamDepartment of Public HealthRotterdamNetherlands

**Keywords:** esophageal cancer, neoadjuvant chemoradiotherapy, esophagectomy, surgery as needed, active surveillance policy

## Abstract

**Background:**

Results from the recent CROSS trial showed that neoadjuvant chemoradiotherapy (nCRT) significantly increased survival as compared to surgery alone in patients with potentially curable esophageal cancer. Furthermore, in the nCRT arm 49% of patients with a squamous cell carcinoma (SCC) and 23% of patients with an adenocarcinoma (AC) had a pathologically complete response in the resection specimen. These results provide a rationale to reconsider and study the timing and necessity of esophagectomy in (all) patients after application of the CROSS regimen.

**Objective:**

We propose a “surgery as needed” approach after completion of nCRT. In this approach, patients will undergo active surveillance after completion of nCRT. Surgical resection would be offered only to those patients in whom residual disease or a locoregional recurrence is highly suspected or proven. However, before a surgery as needed approach in oesophageal cancer patients (SANO) can be tested in a randomized controlled trial, we aim to determine the accuracy of detecting the presence or absence of residual disease after nCRT (preSANO trial).

**Methods:**

This study is set up as a prospective, single arm, multicenter, diagnostic trial. Operable patients with potentially curable SCC or AC of the esophagus or esophagogastric junction will be included. Approximately 4-6 weeks after completion of nCRT all included patients will undergo a first clinical response evaluation (CRE-I) including endoscopy with (random) conventional mucosal biopsies of the primary tumor site and of any other suspected lesions in the esophagus and radial endo-ultrasonography (EUS) for measurement of tumor thickness and area. Patients in whom no locoregional or disseminated disease can be proven by cytohistology will be offered a postponed surgical resection 6-8 weeks after CRE-I (ie, approximately 12-14 weeks after completion of nCRT). In the week preceding the postponed surgical resection, a second clinical response evaluation (CRE-II) will be planned that will include a whole body PET-CT, followed again by endoscopy with (random) conventional mucosal biopsies of the primary tumor site and any other suspected lesions in the esophagus, radial EUS for measurement of tumor thickness and area, and linear EUS plus fine needle aspiration of PET-positive lesions and/or suspected lymph nodes. The main study parameter is the correlation between the clinical response assessment during CRE-I and CRE-II and the final pathological response in the resection specimen.

**Results:**

The first patient was enrolled on July 23, 2013, and results are expected in January 2016.

**Conclusions:**

If this preSANO trial shows that the presence or absence of residual tumor can be predicted reliably 6 or 12 weeks after completion of nCRT, a randomized trial comparing nCRT plus standard surgery versus chemoradiotherapy plus “surgery as needed” will be conducted (SANO trial).

**Trial Registration:**

Netherlands Trial Register: NTR4834; http://www.trialregister.nl/trialreg/admin/rctview.asp?TC=4834 (archived by Webcite at http://www.webcitation.org/6Ze7mn67B).

## Introduction

### Background

Cancer of the esophagus remains a highly lethal malignancy, as reflected by an average overall 5-year survival of 17% [1]. In the Netherlands, the incidence of esophageal cancer resembles the growing trend in Western countries, with an estimated incidence of 15/100,000 for men and 6/100,000 for women [2] and more than 2,500 new cases being diagnosed nationally each year.

At present, surgical resection is still considered the cornerstone of curative treatment for patients eligible with stage cT1b-4aN0-3M0 disease. The reported 5-year survival rate for patients who undergo an esophagectomy ranges from 20% to 50%, but rarely exceeds 35% [3-7]. Esophagectomy is associated with postoperative mortality rates of 1% to 5% in high-volume centers, severe postoperative morbidity, and a substantial impact on the quality of life [8-13]. In order to improve the radicality of surgical resection and the long-term survival after surgical resection, many trials have been performed to study the effect of neoadjuvant chemo and/or radiation therapy [14-17]. One of the largest trials is the recently published chemoradiotherapy for oesophageal cancer followed by surgery study (CROSS trial). This randomized trial compared neoadjuvant chemoradiotherapy (nCRT) plus surgery to surgery alone [18].

During a 5-year period, 366 patients from 5 academic and 2 nonacademic high-volume teaching hospitals in the Netherlands were included in the CROSS trial. Results showed that the addition of nCRT (carboplatin AUC2, paclitaxel 50 mg/m^2^, and 41.4 Gy of concurrent radiotherapy) to surgery significantly increases long-term survival as compared to surgery alone. Median overall survival of patients who received nCRT plus surgery was 49 months, compared to 24 months for those who received surgery alone, and the 3-year overall survival was superior in the nCRT arm (hazard ratio (HR) = 0.66; 95% confidence interval (CI) 0.50-0.87; *P*=.003). Therefore, nCRT plus surgery is now considered the therapy of choice in the Netherlands and several other countries for potentially curable esophageal cancer (cT2-3N0-3M0 and cT1N1-3M0, according to the UICC TNM classification [19]). In subsequent analyses of secondary endpoints of the CROSS trial an interesting observation was made. In the nCRT arm, 49% of patients with a squamous cell carcinoma (SCC) and 23% of patients with an adenocarcinoma (AC) had a pathologically complete response (pCR) in the resection specimen (ie, no viable tumor cells were found, neither at the site of the primary tumor nor in the resected regional lymph nodes, as determined by conventional histological examination) [18]. Therefore, these results provide a rationale to reconsider and study the timing and necessity of standard esophagectomy in patients after application of the CROSS regimen.

### Objective

We propose a “surgery as needed” approach after completion of nCRT for carcinoma of the esophagus. In this surgery as needed approach, patients will undergo active surveillance after completion of nCRT. Surgical resection would be offered only to those patients in whom a locoregional recurrence is highly suspected or proven, in the absence of any signs of distant dissemination. Such an organ-preserving strategy would clearly have great advantages. Postoperative mortality and severe morbidity (grade ≥3 according to the Clavien-Dindo classification [20]) after esophagectomy in the Netherlands is 5% and 60%, respectively. Thus, a nonsurgical treatment strategy in patients with a clinically complete response after nCRT, theoretically saves 5% mortality and 60% severe morbidity in this patient group. Moreover, this approach might improve quality of life and might lead to a reduction in health care costs. However, this surgery as needed approach is only favorable if long-term survival would be comparable to that of the trimodality approach comprising nCRT followed by standard surgery. Before a surgery as needed approach can be tested in a randomized trial, we aim to determine the feasibility of accurate detection of residual disease after chemoradiotherapy through a surgery as needed in oesophageal cancer patients study (preSANO trial).

The aim of this present prospective, multicenter, and diagnostic preSANO trial is to determine the accuracy by which we can detect the presence or absence of residual disease after nCRT. The results of this trial will inform us about the percentage of patients with a clinically complete response after nCRT and will help to estimate the number of patients needed for a subsequent randomized controlled trial. The future so-called “SANO trial” will randomize patients into 2 strategy groups: (1) nCRT plus surgery, and (2) nCRT followed by an active surveillance.

## Methods

### Study Design

The preSANO trial is a prospective, multicenter, diagnostic trial including 120 patients, using a single arm. Five high-volume centers in the Netherlands are currently participating in this study: Erasmus Medical Center, Rotterdam; Academic Medical Center, Amsterdam; University Medical Center, Utrecht; Catharina Cancer Center, Eindhoven; and Atrium Medical Center, Heerlen. The study has been approved by the medical ethics committee (MEC) of the Erasmus Medical Center (MEC2013-211) and has been registered in the Netherlands Trial Register (NTR4834).

### Study Population

**Figure 1:**
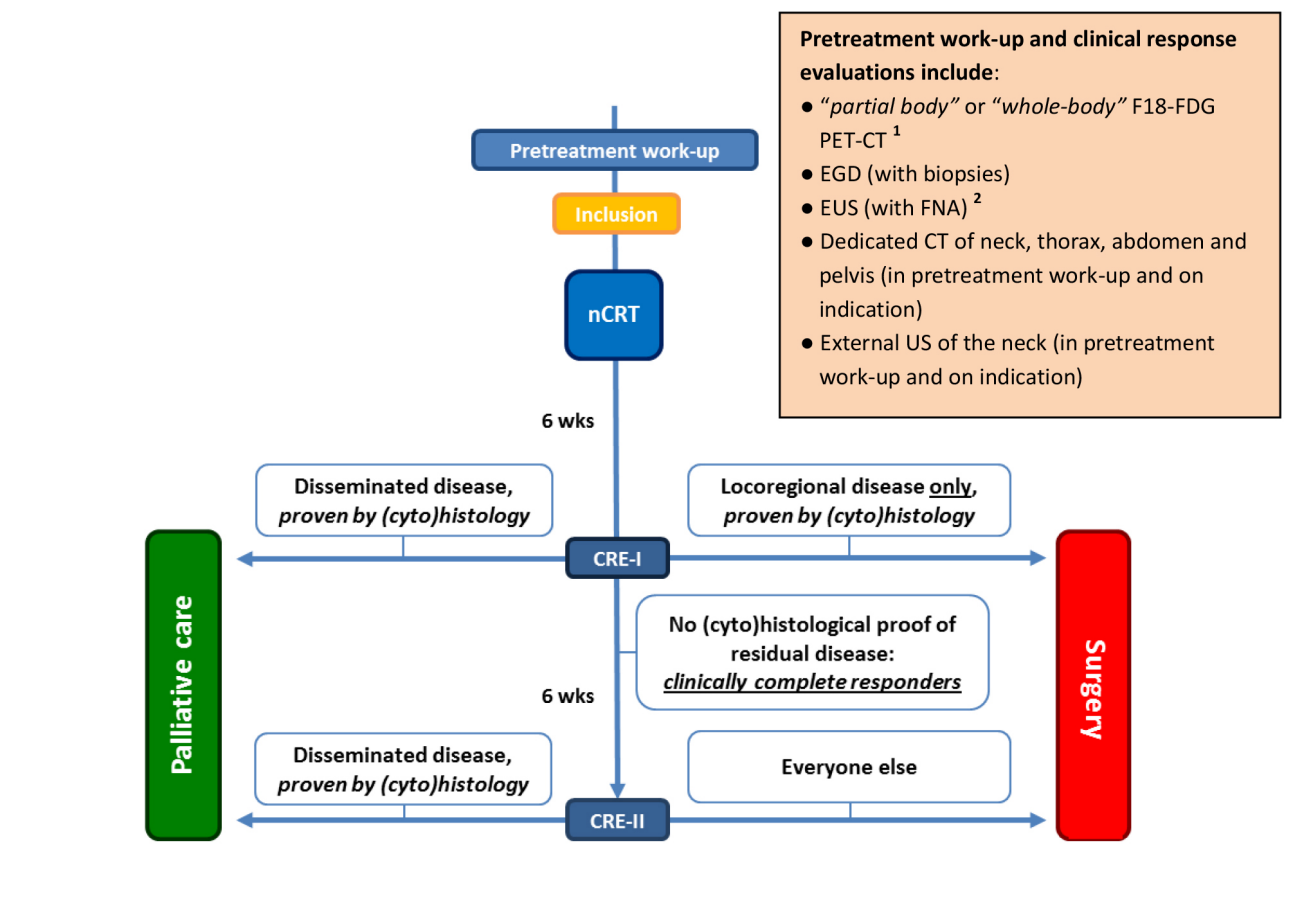
Study algorithm. 1. During the pretreatment workup, it suffices when a “partial body” F18-FDG PET-CT of the esophagus will be performed (to test for avidity of the primary lesion); if it is preferred to make a “whole-body” PET-CT not only after, but also before the neoadjuvant chemoradiotherapy in order to detect distant metastases at an earlier stage, the indication for performing an external US with FNA of the neck can be limited to those patients who have a suspected lymph node on the PET-CT [23]. In the period after neoadjuvant therapy, 1 whole-body F18-FDG PET-CT will be performed either at CRE-I (for the clinically noncomplete responders) or at CRE-II (for the clinically complete responders at CRE-I). 2. EUS with FNA of suspected lymph nodes only during CRE-II, not during CRE-I. CRE: clinical response evaluation; CT: computed tomography; EUS: endoscopic ultrasonography; FNA: fine-needle aspiration; nCRT: neoadjuvant chemoradiotherapy; EGD: esophagogastroduodenoscopy; PET: positron-emission tomography; US: ultrasonography.

We plan to include individuals from a population of operable patients with potentially curable SCC or AC of the esophagus or esophagogastric junction. All patients who are planned to undergo nCRT according to the CROSS regimen [18] followed by surgical resection are eligible to participate. Patients with dementia or altered mental status prohibiting the understanding and giving of informed consent will be excluded from participation in this study. Patients will undergo conventional pretreatment selection (including at least a “partial body” F18-FDG positron emission tomography-computed tomography (PET-CT) to assess the avidity of the primary tumor process; Figure 1 and Table 1).

**Table 1:** Study algorithm.

Parameter	Pretreatment	First clinical response evaluation (CRE-I)	Second clinical response evaluation (CRE-II)
History, physical examination	X	X	X
Performance status	X	X	X
Hematology^a^	X		
eGFR	X		
Biochemistry^b^	X		
Endoscopy + (random) biopsies	X	X	X
Radial EUS^c^	X	X	X
Linear EUS (+FNA)^d^	X		X
CT of neck, thorax, abdomen, and pelvis	X		
PET-CT	X “partial body”	X^g^“whole body”	X^h^“whole body”
Pulmonary function tests	X		
Bronchoscopy^e^	X		
ECG	X		
Toxicity^f^	Baseline		

^a^Hematology: CBC, differential

^b^Biochemistry: serum protein, albumin, magnesium, electrolytes, serum creatinine, bilirubin, alkaline phosphatase, AST, and pregnancy test if indicated at baseline only

^c^Radial EUS: with measurement of maximum tumor thickness and area

^d^Linear EUS: with FNA of any suspected lymph nodes

^e^Bronchoscopy: when tumor is located above the carina and when there is suspicion for invasion of the tracheobronchial tree

^f^Toxicity: to be evaluated after each cycle (incidence and grade according to CTC toxicity scale)

^g^PET-CT: during CRE-I, after EGD and EUS, only for clinically noncomplete responders to exclude disseminated disease

^h^PET-CT: during CRE-II, prior to EGD and EUS, for all patients (all were clinically complete responders during CRE-I) to guide EGD and EUS in targeting suspected locoregional lesions and to exclude disseminated disease

### Study Algorithm

#### Overview

All included patients will receive nCRT according to the CROSS protocol (carboplatin, paclitaxel, and concurrent radiotherapy) [18]. Patients will be reevaluated either once or twice before undergoing surgical resection during clinical response evaluations (CRE). The aim of these CREs will be to identify those patients in whom residual and/or disseminated disease is present.

#### CRE-I

The first CRE (CRE-I) will be performed 4-6 weeks after completion of chemoradiotherapy (Figure 1). During CRE-I, all patients will undergo esophagogastroduodenoscopy (EGD) with registration of endoscopic images for future reference and biopsies of any suspected lesions, including mucosal biopsies at the site of the primary tumor (1 regular biopsy per centimeter in each of the 4 quadrants), radial endoscopic ultrasonography (EUS) for measurement of maximal tumor thickness and area, and linear EUS. Patients with histological evidence of locoregional residual disease, but without evidence of disseminated disease, will be offered immediate surgical resection. These patients have no clear benefit from postponement of surgical resection and should therefore have no delay according to current recommendations. Patients without histological evidence of locoregional residual disease and without disseminated disease will be considered to be *clinically complete responders* and will be offered a postponed surgical resection. In these patients a surgical resection will be postponed for an additional 6-8 weeks, allowing patients more time to reach a better condition for surgery.

#### CRE-II

In the week preceding the planned postponed surgical resection, a second clinical response evaluation (CRE-II) will be scheduled. CRE-II will be performed only in patients who were considered to be clinically complete responders (ie, no viable tumor found) at CRE-I. CRE-II will consist of a PET-CT (standard for all patients at CRE-II and only for tumor-positive patients at CRE-I), an EGD with registration of endoscopic images for future reference, and biopsies of any suspected lesions, including (random) mucosal biopsies at the site of the primary tumor, radial EUS for measurement of maximal tumor thickness and area, and linear EUS plus fine-needle aspiration (FNA) of PET-positive lesions and/or suspected lymph nodes.

An important difference between CRE-I and CRE-II will be that during CRE-I clinically complete responders will be offered a postponed surgical resection, whereas after CRE-II both locoregionally complete and noncomplete responders will be advised to undergo a surgical resection (Figure 1). In other words, all patients who are considered clinically complete responders at CRE-I and are therefore allowed to postpone their surgery by an additional 6-8 weeks, will undergo CRE-II followed by the postponed surgical resection, irrespective of the locoregional findings during CRE-II. The diagnostic results from CRE-II will later be compared with results from both CRE-I and the final pathological analysis of the resection specimen. However, patients with (cyto)histological evidence of disseminated disease during CRE-I or CRE-II will be excluded from further curative therapy and will be referred for palliative care.

If after CRE-II the planned operation is postponed for more than 4 weeks (eg, because the patient has not yet sufficiently recovered from the nCRT), a CRE-III (comparable to CRE-II) will be performed 1 week before the (further) postponed operation.

#### Surgery

Surgical resection will be attempted immediately after CRE-I only in those patients who present at CRE-I with histologically proven residual disease after completion of nCRT, without any signs of disseminated disease. All other patients will undergo surgical resection after CRE-II in the absence of distant metastases.

A transthoracic esophageal resection or a transhiatal approach can be performed, depending on both patient characteristics and local expertise and preference. Both open and minimally invasive techniques are allowed.

A wide local excision including the regional lymph nodes is carried out in both techniques, including a standard dissection of the lymph nodes around the coeliac axis. The continuity of the digestive tract will preferably be restored by a gastric tube reconstruction or, if required, by a colonic interposition.

At least 15—but preferably 23 or more—lymph nodes should be aimed to be removed in every patient since it has been shown that long-term survival is maximized with the removal of at least 23 nodes [21]. Moreover, the risk of understaging the tumor in these patients should be minimized. If an insufficient number of nodes is removed, the patient might be erroneously staged as ypN0, while in fact ypNpos nodes have been left in situ (stage migration).

#### Pathology

All resection specimens will be revised centrally by 2 independent expert pathologists, using a standard protocol. In case of a discordant outcome, the specimens will be reviewed by a third independent expert pathologist. A final diagnosis will be made only if at least 2 pathologists agree. Also, all the CRE-II biopsies of patients who were considered negative at CRE-II, but who had more than 10% residual tumor in their resection specimen will be revised centrally following the same strategy. In these specimens special attention will be given to the effects of the preoperative chemoradiation (ie, tumor reduction and therapy effects). The lymph node dissection should contain at least 15—but preferably 23 or more—nodes derived from both mediastinum and upper abdomen, which are essential for correct ypTNM staging. The resection margins, especially the circumferential margin, will be evaluated with a 1 mm cutoff point for vital tumor. This implies that the tumor-free margin should be larger than 1 mm in order to be classified as R_0_. If vital tumor is present at 1 mm or less from the surgical resection margin, it is considered microscopically positive (R_1_).

#### Interim Analysis

An interim analysis will be performed by an independent safety committee after a total inclusion of 60 patients in order to carefully monitor serious complications during CRE-I and CRE-II and to assess the achieved radicality of the performed operations.

### Main Study Parameter/Endpoint

The main study parameter in this study is the correlation between the clinical response assessment during CRE-I and CRE-II and the final pathological response in the resection specimen as measured by the modified tumor regression grading (TRG) system of Chirieac [22]: no residual carcinoma (TRG1), 1%-10% residual carcinoma (TRG2), 11%-50% residual carcinoma (TRG3), 51%-100% residual carcinoma (TRG4) [22].

We propose that in this study TRG2 residual tumors may be missed as long as we expect them to be detectable reliably as soon as they have outgrown from TRG2 to TRG3-4 during follow-up. The risk that TRG2 residual tumors will lead to irresectability in the short-term is likely to be small/negligible. However, we do propose that TRG3 and TRG4 residual tumors should be detected without further delay in order to prevent short-term loss of resectability and to minimize the risk of long-term distant disease dissemination. The validity of these assumptions can only be determined in a future SANO trial, in which an active surveillance strategy will be compared with standard surgery in all patients after nCRT.

### Statistical Analysis

#### Sample Size Calculation

**Figure 2:**
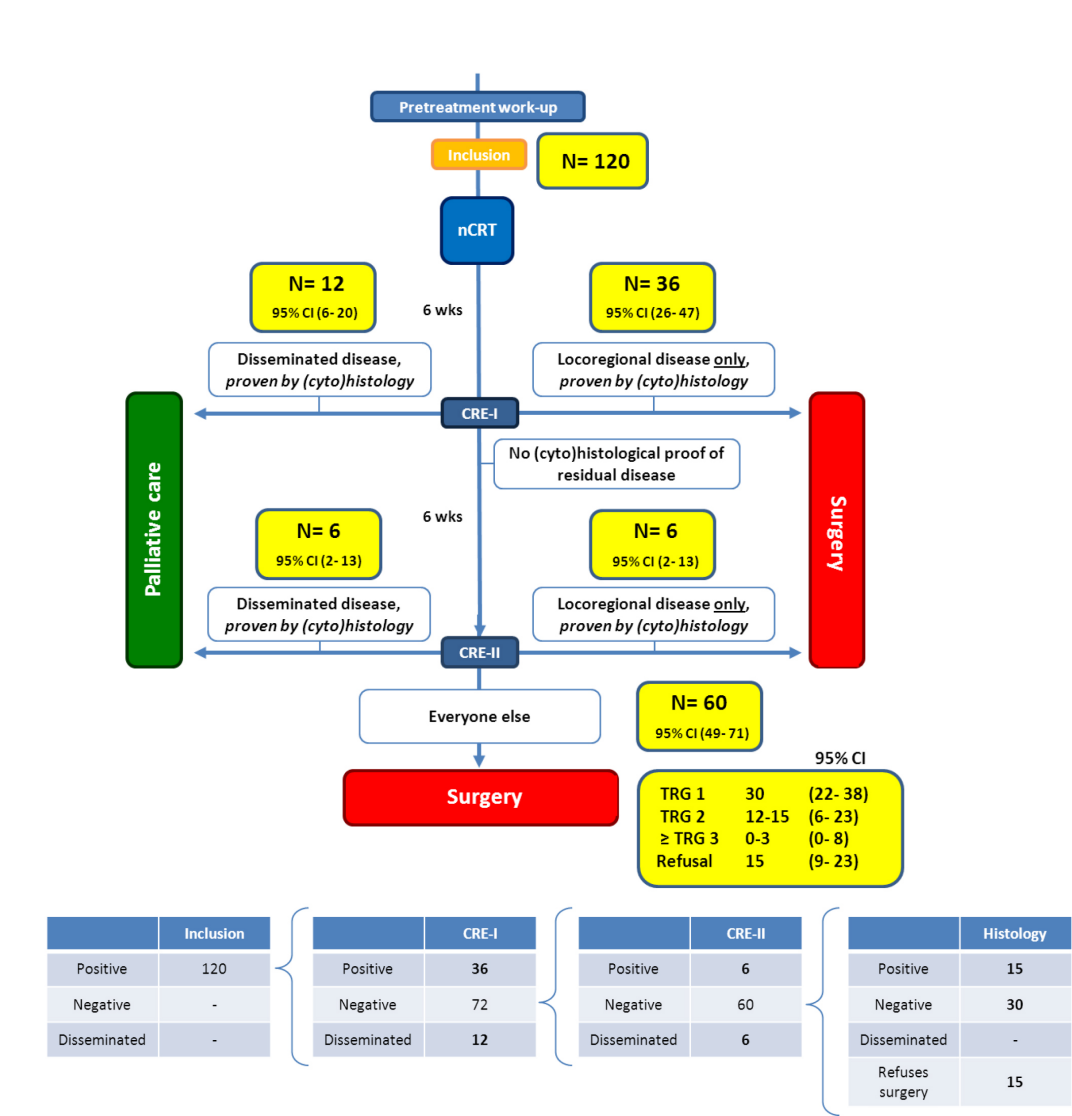
Expected distribution of patients (based partly on CROSS trial data). All numbers are based on an inclusion of 120 patients. CI: confidence interval; CRE: clinical response evaluation; nCRT: neoadjuvant chemoradiotherapy; N: number of patients; TRG: tumor regression grade, as measured by the modified TRG system of Chirieac. Of the 45 patients who will undergo a postponed resection following CRE-II, 15 patients are expected to have a pathologically incomplete response (at least TRG2).

As was seen in the previous CROSS trial approximately 40% of the included patients will have TRG3 or TRG4 residual tumor in the resection specimen [18]. With a total inclusion of 120 patients, approximately 45 patients will have TRG3 or TRG4 residual tumor. We consider 45 patients a sufficiently large sample for determining the accuracy of individual and/or combined diagnostic tests. In order to estimate the distribution of 120 patients planned to be included, data were used from the CROSS trial as indicated in Figure 2. Furthermore, several assumptions were made:

- We assume that during CRE-I clinically complete responders will comprise patients with TRG1 or TRG2 (as taken from the pathological response data of the CROSS trial), whereas clinically noncomplete responders will be patients with TRG3 or TRG4.
- The percentage of patients with SCC and AC with TRG1 or TRG2 in the CROSS trial was 78% and 57%, respectively. This means that approximately 60% of included patients are expected to have negative (cyto)histology at CRE-I.
- In a trial by Blom et al [23], approximately 10% of patients who were reevaluated by PET-CT after completion of nCRT had newly discovered disseminated disease. We assume that there will be fewer newly found disseminated disease with positive (cyto)histology at CRE-II because a number of these patients are expected to be discovered during CRE-I.
- We assume that approximately 25% of clinically complete responders will refuse to undergo the postponed resection and choose to undergo an active surveillance strategy if no alarming results are found during CRE-II.

These calculations indicate that approximately 60 patients will show a clinically complete response after combined diagnostic investigations during CRE-I and CRE-II (including EUS-FNA with tumor thickness measurements and PET-CT). Of these, approximately 15 patients will refuse to undergo surgery and will undergo active surveillance and approximately 30 patients will have a pCR (TRG1). The 15 remaining patients are expected to have residual disease, of whom approximately 12 patients will have TRG2 residual tumor and approximately 3 patients will have TRG3 or TRG4 residual tumor. As we proposed above, TRG2 residual tumors may be missed. Therefore, we expect that approximately 3 patients with clinically relevant residual disease (TRG3 or TRG4) will be missed.

In case of unexpected aberrant distribution of patients in the preSANO trial that leads to decreased TRG3 and TRG4 rates, results of the first 120 patients will be analyzed following the present protocol. If these results are promising but do not reach statistical significance, possibly due to a lack of power, inclusion of extra patients will be considered. If inclusion of extra patients is desirable, the protocol will be amended and assessed by the medical ethics committee.

#### Data Analysis

The clinical response evaluation will consist of different diagnostic modalities. Results of each diagnostic modality will be presented as categorical or continuous data, depending on the outcome measure of each diagnostic modality. These results will be correlated to the (categorical) tumor regression grading in the resection specimen using a Chi-square-based test (categorical-categorical) or a 1-way ANOVA test (continuous-categorical) with post-hoc testing.

## Results

The first patient was enrolled on July 23, 2013, and results are expected in January 2016.

## Discussion

The uniqueness of this study lies in the prospective evaluation of a sufficiently large number of patients, using multiple diagnostic modalities on different time points. Although (cyto)histological assessment of biopsies and/or FNAs is the most objective parameter, several studies have shown that the response to nCRT is reflected by tumor size or volume as assessed by EUS [24-27]. The rationale to include a second clinical response evaluation before a planned surgical resection is to allow for a comparison between multiple measurements and to increase the chance of detecting residual and/or disseminated disease. It is expected that during CRE-II (due to an extended time period from the end of nCRT) the F18-FDG PET-CT signal will have a more favorable signal-to-noise ratio than has been described previously [28-33] because after 12 weeks the artefacts due to radiation-induced inflammation are expected to have largely dissolved. This allows for identification of suspected lymph nodes to be targeted by FNA during CRE-II.

The reason to include patients with SCC as well as patients with AC in the preSANO trial is that the CROSS regimen has been shown to be effective in both groups of patients. The pCR rates of 49% in patients with SCC and 23% in patients with AC in the CROSS trial provide a rationale for a SANO approach in both histological subtypes. Furthermore, together with the low frequency of toxic effects of the CROSS regimen (91% received the full treatment regimen of nCRT), these high pCR rates advocate the use of the relatively low dose of 41.4 Gy radiotherapy [18].

Although we have not yet clearly shown that we are able to detect a clinically threatening residual cancer 4-6 weeks after nCRT, there are several arguments why it is not deemed necessary to do so before we delay the planned surgical resection with an additional 6-8 weeks. Recently, it was shown that prolonged time to surgery after nCRT up to at least 12 weeks had no effect on disease-free and overall survival (HR=1.00 and HR=1.06 per additional week, *P*=.976 and *P*=.139, respectively). Moreover, prolonged time to surgery increased the probability of pCR in the resection specimen (odds ratio = 1.35 per additional week of time to surgery, *P*=.0004) [34]. Comparable results have been published by other groups [35,36].

Postoperative mortality and severe morbidity (grade ≥3 according to the Clavien-Dindo classification [20]) after esophagectomy in the Netherlands is 5% and 60%, respectively. Thus, a nonsurgical treatment strategy in patients with a clinically complete response after nCRT theoretically saves up to 5% mortality and 60% severe morbidity in this patient group. Moreover, this approach might improve quality of life and might lead to a reduction in health care costs. Therefore, we will consider this study as successful when the results of the combined diagnostic modalities lead to a maximum percentage of clinically false-negative TRG3 and TRG4 tumors of twice the postoperative mortality (ie, 10%). If more than 10% of TRG3 or TRG4 tumors are missed, the SANO trial will be reconsidered.

If the preSANO trial shows that TRG3 and TRG4 residual tumor can be predicted reliably, a randomized trial comparing nCRT plus standard surgery versus chemoradiotherapy plus surgery as needed in oesophageal cancer patients (the SANO trial) will be conducted. Hopefully, this SANO trial will result in an organ-preserving treatment strategy for a selected group of patients and therefore reduce treatment related morbidity and mortality, improve quality of life, and lead to a reduction in health care costs.

## Multimedia Appendix 1

The trial is funded by amongst others the Koningin Wilhelmina Fonds Kankerbestrijding (KWF, Dutch Cancer Society) and has been reviewed by external reviewers from the KWF. They assigned the study an A-status, indicating that funding of this project is of the highest priority.

## Abbreviations

AC: adenocarcinoma
CI: confidence interval
CRE: clinical response evaluation
CROSS: chemoradiotherapy for oesophageal cancer followed by surgery study [18]
CT: computed tomography
EGD: esophagogastroduodenoscopy
EUS: endoscopic ultrasonography
FNA: fine-needle aspiration
HR: hazard ratio
MEC: medical ethics committee
nCRT: neoadjuvant chemoradiotherapy
NTR: Netherlands Trial Register
pCR: pathologically complete response
PET: positron-emission tomography
SANO: surgery as needed approach in oesophageal cancer patients
SCC: squamous cell carcinoma
TRG: tumor regression grading
