# Supplementary material for: Accuracy of Detecting Residual Disease After Cross Neoadjuvant Chemoradiotherapy for Esophageal Cancer (preSANO Trial): Rationale and Protocol
Source: JMIR Res Protoc. 2015 Jun 29;4(2):e79. doi: 10.2196/resprot.4320 (PMC4526968; doi:10.2196/resprot.4320)
Supplement: Supplementary file 1 [file resprot_v4i2e79_app1.pdf]

KWF KANKER BESTRIJDING

ADVIES

WETENSCHAPPELIJKE RAAD VAN KWF KANKERBESTRIJDING

Datum: 2 december 2014

Advies over project:
Accuracy of detecting residual disease after neoadjuvant chemoradiotherapy for oesophageal cancer (pre-SANO trial)

Projectnummer: EMCR 2014-7430
Projectleiders: Prof. dr. J.J.B. van Lanschot
Dr. B.P.L. Wijnhoven

Beoordeling op basis van kwaliteit: A
(zie bijgevoegd informatieblad)

Toelichting op de beoordeling:

Referent 1:
De vragen en opmerkingen van de vorige aanvraag zijn goed beantwoord.

Referent 2:
Originaliteit van de studie:
Vorige keer al benoemd, originele en belangrijke onderzoeksvraag.
Belang van de studie:
Zeer duidelijk dat het weglaten van de operatie veel kan betekenen voor de patiënt. Het accuraat kunnen voorspellen van residual disease o.b.v. diagnostische modaliteiten is een belangrijke eerste vereiste.
Haalbaarheid van de studie:
Accrual is al gaande en verloopt goed. Inmiddels ook een extra centrum geopend.
Uitvoerbaarheid van de studie:
Omdat het onderzoek al gaande is wordt een goede indruk verkregen van de uitvoerbaarheid van de studie.
Methodologie:
Ondanks afwezigheid van formele sample size berekening laten de onderzoekers nu veel inzichtelijker zien wat zij aan resultaten verwachten en wat zij nog acceptabel vinden als uitgangspunt om aan een gerandomiseerde studie te gaan beginnen.
De onderzoekers verwachten bij 3 van de 45 patienten met TGR 3 of 4 residual disease dat ze op basis van de diagnostiek een klinisch complete respons laten zien. Dit is een percentage van 7% maar het 95% betrouwbaarheidsinterval loopt tot 19%. Dus bij deze sample size neem je het risico op een maximum van 19% ipv 10%.
Bij het geblindeerd beoordelen van de diagnostische modaliteiten bedoelde ik ook dat de verschillende diagnostische modaliteiten (PET-CT, gastrosopie, ultrasonografie) onafhankelijk van elkaar beoordeeld zouden worden.

Patiënten Advies Commissie Onderzoek (PACO)

Betrokkenheid patiënten

Medisch congres nationaal en internationaal, voorts in med.wet.tijdschrift (basis voor een vervolg prospectief (inter) nationaal onderzoek.

Patienten Informatie Formulier (PIF)

- Detecteren: op te sporen, graag eenvoudig woord gebruik (bld 1 laatste zin).
- Ad standaard behandeling, pag.8 tekening is erg zwart getekend,daarom moeilijk zichtbaar/leesbaar voor de patiënt.

December 2014

INFORMATIEBLAD

BEOORDELINGSPROCEDURE KLINISCHE STUDIES

In dit informatieblad treft u informatie aan over de beoordelingsprocedure voor subsidieaanvragen voor datamanagement voor klinische studies en startsubsidies voor studiegroepen.

De projecten zijn beoordeeld door de Wetenschappelijke Raad van KWF Kankerbestrijding (WR). Projecten, inclusief CRFs, zijn beoordeeld op wetenschappelijke kwaliteit, maar ook op uitvoerbaarheid, methodologie, haalbaarheid en belang voor de kankerbestrijding. Indien gewenst kan de WR het oordeel van externe deskundigen op het betreffende onderzoeksgebied bij haar beoordeling betrekken. Naast de inhoudelijke beoordeling is er ook gekeken naar de financiële inbedding van het project. Projecten zonder externe financiering krijgen prioriteit t.o.v. studies die zijn ondersteund door bijvoorbeeld de farmaceutische industrie.

De WR heeft voor de subsidie aanvraag een kwaliteitsoordeel vastgesteld binnen het onderstaande classificatiesysteem. Tevens is door de WR aangegeven welke kwaliteitsklassen naar zijn mening voor KWF-financiering in aanmerking kunnen komen. Uiteraard betekent dit niet dat alle aanvragen binnen deze kwaliteitsklassen ook daadwerkelijk kunnen worden gefinancierd.

A	projecten die met hoge prioriteit voor financiering in aanmerking komen.
B1, B2	projecten die bij voldoende middelen voor financiering in aanmerking kunnen komen.
R	projecten die in de huidige vorm niet voor financiering in aanmerking komen, maar waarvan de WR adviseert om na aanpassing aan commentaren van referenten en het advies van de WR opnieuw bij KWF Kankerbestrijding in te dienen.
C	projecten die niet voor financiering in aanmerking komen.

Indien een projectleider van mening is dat in de commentaren van de referenten feitelijke onjuistheden staan en dat deze van een dusdanig gewicht zijn dat zij de Raad mogelijk naar een onjuist kwaliteitsoordeel hebben geleid, dan kan de projectleider binnen 10 weken na verzending van het advies schriftelijk bezwaar aantekenen. U kunt uw bezwaar sturen naar:

KWF Kankerbestrijding
Team Bestedingen
Postbus 75508
1070 AM AMSTERDAM
bestedingen@kwf.nl
